# Supplementary figures and images for: Zoledronic acid promotes osteoclasts ferroptosis by inhibiting FBXO9-mediated p53 ubiquitination and degradation
Source: PeerJ. 2021 Dec 16;9:e12510. doi: 10.7717/peerj.12510 (PMC8684721; doi:10.7717/peerj.12510)

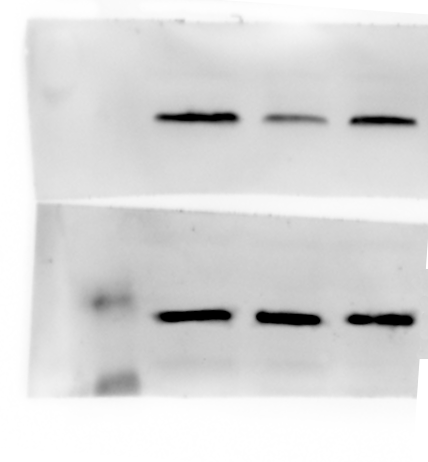

Supplement: Supplemental Information 2 [file peerj-09-12510-s002.zip › WB汇总/fig-5.tif]

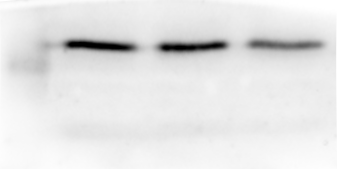

Supplement: Supplemental Information 2 [file peerj-09-12510-s002.zip › WB汇总/Figrue4 FBOX9.tif]

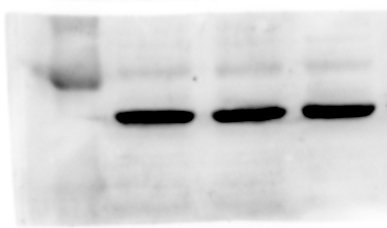

Supplement: Supplemental Information 2 [file peerj-09-12510-s002.zip › WB汇总/Figrue4 GAPDH.tif]

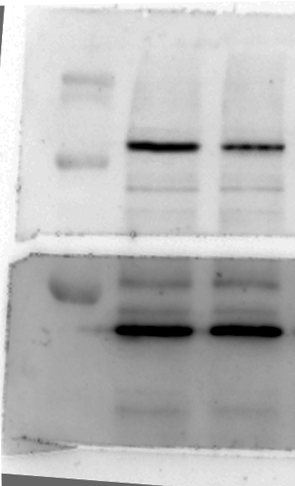

Supplement: Supplemental Information 2 [file peerj-09-12510-s002.zip › WB汇总/Figur3D left.tif]

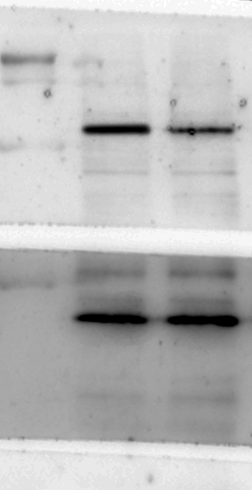

Supplement: Supplemental Information 2 [file peerj-09-12510-s002.zip › WB汇总/Figur3D right.tif]

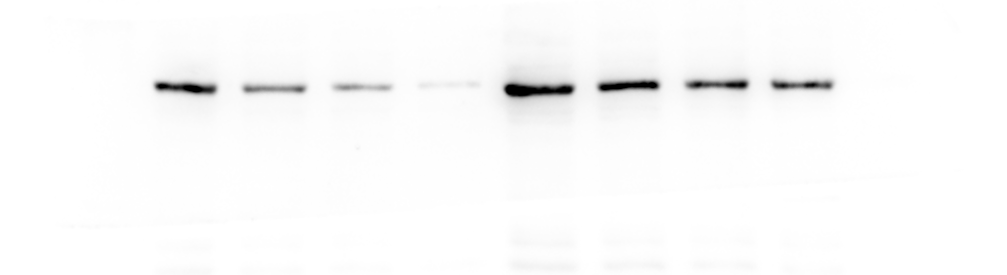

Supplement: Supplemental Information 2 [file peerj-09-12510-s002.zip › WB汇总/Figure 6e P53.tif]

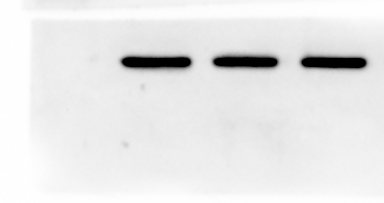

Supplement: Supplemental Information 2 [file peerj-09-12510-s002.zip › WB汇总/Figure 6F GADPH.tif]

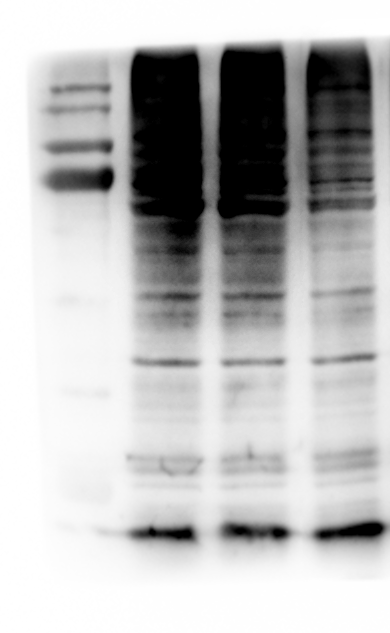

Supplement: Supplemental Information 2 [file peerj-09-12510-s002.zip › WB汇总/Figure 6F UB.tif]

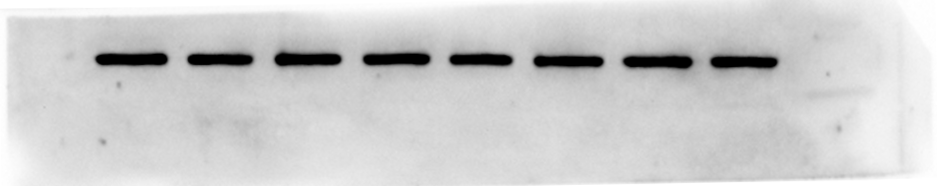

Supplement: Supplemental Information 2 [file peerj-09-12510-s002.zip › WB汇总/Figure6e GAPDH.tif]

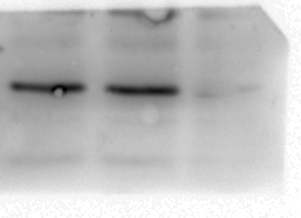

Supplement: Supplemental Information 2 [file peerj-09-12510-s002.zip › WB汇总/IP-FBXO9 IB FBXO9.tif]

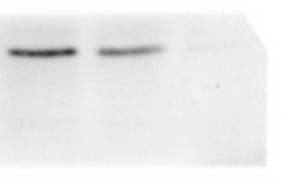

Supplement: Supplemental Information 2 [file peerj-09-12510-s002.zip › WB汇总/IP-FBXO9 IB P53.tif]

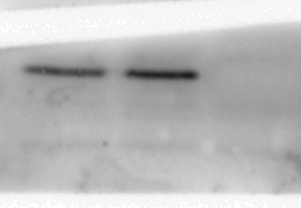

Supplement: Supplemental Information 2 [file peerj-09-12510-s002.zip › WB汇总/IP-P53 IB FBXO9.tif]

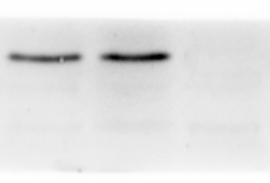

Supplement: Supplemental Information 2 [file peerj-09-12510-s002.zip › WB汇总/IP-P53 IB P53.tif]
